# Expression of functionally active sialylated human erythropoietin in plants

**DOI:** 10.1002/biot.201200363

**Published:** 2013-01-17

**Authors:** Jakub Jez, Alexandra Castilho, Josephine Grass, Karola Vorauer-Uhl, Thomas Sterovsky, Friedrich Altmann, Herta Steinkellner

**Affiliations:** 1 Department of Applied Genetics and Cell Biology, University of Natural Resources and Life SciencesMuthgasse, Vienna, Austria; 2 Department of Chemistry, University of Natural Resources and Life SciencesMuthgasse, Vienna, Austria; 3 Department of Biotechnology, University of Natural Resources and Life SciencesMuthgasse, Vienna, Austria; 4 PolymunScientificKlosterneuburg, Austria

**Keywords:** Erythropoietin, Glycoengineering, Plants, Purification, Sialylation

## Abstract

Recombinant human erythropoietin (rhEPO), a glycohormone, is one of the leading biopharmaceutical products. The production of rhEPO is currently restricted to mammalian cell expression systems because of rhEPO's highly complex glycosylation pattern, which is a major determinant for drug-efficacy. Here we evaluate the ability of plants to produce different glycoforms of rhEPO. cDNA constructs were delivered to *Nicotiana benthamiana* (*N. benthamiana*) and transiently expressed by a viral based expression system. Expression levels up to 85 mg rhEPO/kg fresh leaf material were achieved. Moreover, co-expression of rhEPO with six mammalian genes required for *in planta* protein sialylation resulted in the synthesis of rhEPO decorated mainly with bisialylated *N*-glycans (NaNa), the most abundant glycoform of circulating hEPO in patients with anemia. A newly established peptide tag (ELDKWA) fused to hEPO was particularly well-suited for purification of the recombinant hormone based on immunoaffinity. Subsequent lectin chromatography allowed enrichment of exclusively sialylated rhEPO. All plant-derived glycoforms exhibited high biological activity as determined by a cell-based receptor-binding assay. The generation of rhEPO carrying largely homogeneous glycosylation profiles (GnGnXF, GnGn, and NaNa) will facilitate further investigation of functionalities with potential implications for medical applications.

## 1 Introduction

Erythropoietin (EPO) is a glycoprotein mainly produced in the adult kidney, and was initially highlighted for its action on the hematopoietic system [1, 2]. EPO is also expressed in several non-hematopoietic tissues, where it protects from apoptosis and inflammation due to hypoxia, toxicity or injury [3, 4].

The human erythropoietin (hEPO) gene was first cloned in 1985 [5, 6]. Recombinant hEPO (rhEPO) has been available as a drug for over 25 years and is mainly used for treating anemia. In sports, its misuse has been suspected among athletes for many years [7]. rhEPO has a legitimate worldwide market of about 5 billion dollars per year, making it one of the leading biopharmaceutical products [8].

Active hEPO consists of a single 166 amino-acid polypeptide chain with three *N*-glycosylation sites at Asn^24^, Asn^38^, and Asn^83^, and one *O*-glycosylation site at Ser^126^. All sites are extensively glycosylated [9, 10] and the critical influence of this posttranslational modification for drug efficacy has been reported [11, 12]. Consequently, the oligosaccharide structures of native and rhEPO have been studied extensively. The hormone has been shown to have a large *N*-glycan microheterogeneity but an otherwise homogeneous protein backbone. Native and rhEPOs, isolated from urine and Chinese hamster ovary (CHO) cells, respectively, carry large amounts of elongated and branched structures (i.e. sialylated bi-, tri-, and tetra-antennary oligosaccharides). Native hEPO isolated from serum, which presents the physiologically active form of the hormone, exhibits in general the same type of *N*-linked oligosaccharides in terms of branching patterns as CHO-derived rhEPO but the relative amounts of the individual structures are different. A major difference is the presence of relatively large amounts of non-sialylated GnGnF structures and the absence of tetra-antennary oligosaccharides in circulatory hEPO [13]. Notably large glycan variations can occur between different individuals' urinary EPO [14]. These observations indicate that *N*-glycans actively contribute to the modulation of hEPO activities in vivo.

The importance of terminal sialic acid for the circulatory half-life of rhEPO is well documented [11, 15, 16], and many investigations have concentrated on enhancing the presence of this carbohydrate formation [17, 18]. Indeed, hyper-sialylated rhEPO with a prolonged half-life and subsequently enhanced drug efficacy has been reported [12]. Relatively little is known about the impact of other glycoforms. However, increased understanding is desirable in view of the large *N*-glycan heterogeneity of hEPO and the recent findings of the non-erythropoietic activities of the hormone. Accordingly, it would be extremely valuable to establish an rhEPO with a defined *N*-glycosylation pattern for future research.

Plants are a suitable alternative platform for the expression of complex human recombinant proteins. In recent years the ability to modulate the plant *N*-glycosylation profile toward human-like structures and thus alter the in vivo activities of therapeutic proteins has attracted great attention [19, 20]. Previous attempts to produce rhEPO in plants have resulted in the generation of a recombinant hormone exhibiting in vitro activity [21–24]. Unfortunately, most of these studies largely neglected the *N*-glycosylation status of the recombinant hormone, or reported the presence of unusual oligosaccharide structures [25]. Moreover, rhEPO expressed in tobacco cells failed to show in vivo activity, most probably due to the lack of sialic acid residues [26]. In addition, success in purifying the plant-derived recombinant hormone was also limited.

Here, we conducted a comprehensive study to evaluate the ability of plants to produce different glycoforms of rhEPO. Respective cDNA constructs were delivered to *N. benthamiana* and transiently expressed by a viral-based magnICON® expression system [27]. LC-electro-spray ionization-MS (LC-ESI-MS) used for *N*-glycosylation profiling, revealed that the recombinant hormone carries complex *N*-glycans with and without plant-specific epitopes (GnGnXF and GnGn), depending on whether the expression host is the wild type (WT) or the glycosylation mutant ΔXTFT (a mutant lacking the plant-specific *N*-glycan residues xylose and core α1,3 fucose, [28]). Synthesis of bisialylated rhEPO (NaNa) was achieved by co-expressing the protein with the mammalian genes necessary for *in planta* sialylation [29]. By fusing the short peptide tag ELDKWA to rhEPO, the recombinant hormone was purified via immunoaffinity chromatography and finally sialylated rhEPO was enriched with *Sambucus nigra* agglutinin (SNA) lectin chromatography. Cell-based receptor binding assay demonstrated the expression of a highly active hormone.

## 2 Material and methods

### 2.1 Construction of erythropoietin expression vectors

**Figure 1:**
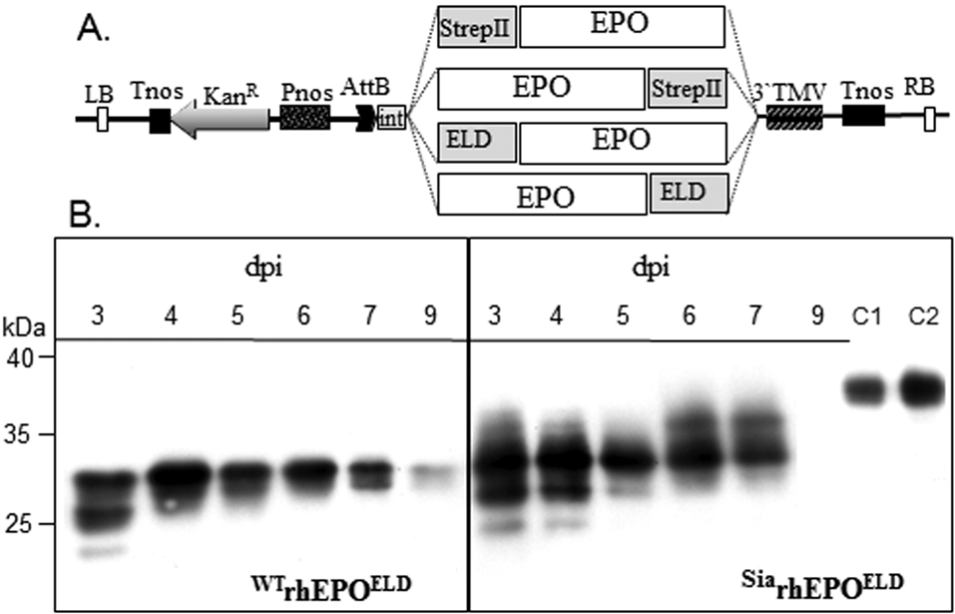
(**A**) Schematic representation of the 3'-modules of the TMV-based magnICON® vectors (pICH21595) used for the expression of the rhEPO. Pnos: nopaline synthase gene promoter; Tnos: nopaline synthase gene terminator; KanR: neomycin phosphotransferase II gene; 3'TMV: 3' untranslated region; AttB: recombination site; int: intron; Strep II: WSHPQFEK peptide sequence that binds specifically to streptavidin; ELD: ELDKWA peptide sequence from the HIV-1-gp41; EPO: plant codon optimized hEPO sequence lacking its native signal peptide sequence (see Section 2); LB: left border; RB: right border. (**B**) Timecourse analysis of rhEPO^ELD^expression in *N. benthamiana*. TSPs (2 μg) from infiltrated leaves were extracted on different days post-infiltration (dpi) and analyzed by western blotting with antibodies against hEPO. ^WT^rhEPO^ELD^:rhEPO^ELD^ expressed in WT plants. ^Sia^rhEPO^ELD^:rhEPO^ELD^ co-expressed with the genes for the mammalian sialic acid pathway [29] in *N. benthamiana* Gal^+^ (mutant lacking β1,2-xylose and core α1,3-fucose, and carrying terminal β1,4-galactose, [30] ). C1 and C2: CHO-derived hEPO (50 and 100 ng, respectively). Data are representative of three independent experimental repeats. The sizes of molecular weight marker proteins are shown in kiloDalton.

A 585-bp fragment containing the cDNA from hEPO (amino acids 28-194), codon optimized for dicot plants and with a C-terminal Strep II tag (WSHPQFEK) was purchased from Mr. Gene GmbH, Regensburg (Germany) (http://www.bionity.com). This sequence was used as a template in a set of PCR reactions using different primer combinations to amplify *Bsa*I-*Bsa*I hEPO fragments with a C- or N-terminal Strep II tag and fragments where the Strep II tag was replaced by the ELDKWA sequence (Table S1 of Supporting information). All four PCR products were digested with *Bsa*I and cloned into a magnICON® tobacco mosaic virus-based 3'-module vector (pICH21595, Bayer BioScience NV Research, Gent, Belgium) containing two *Bsa*I sites designed for directional cloning of the target gene [27]. The resulting vectors were named ^Strep^rhEPO, rhEPO^Strep^, ^ELD^rhEPO, and rhEPO^ELD^ (Fig. 1A). The 5'-module (pICH20999) includes the signal peptide from the barley α-amylase sequence to direct proteins to the secretory pathway. A binary vector (pICH14011) expressing the recombinase was used to allow *in planta* assembly of the two viral modules.

### 2.2 Plant material and transient protein expression

WT *N. benthamiana* plants, the glycosylation mutants ΔXTFT (lacking plant-specific *N*-glycosylation, i.e. β1,2-xylose and core α1,3-fucose, [28]) and Gal^+^ (synthesizing β1,4-galactosylated structures, [30]), were grown at 22°C with a 16-h light: 8-h dark photoperiod. Four to five-week-old plants were used for agroinfiltration experiments as described previously [30]. To express the rhEPO, the 3'-magnICON® vector containing rhEPO cDNA was co-infiltrated with the corresponding 5'-vector carrying the signal peptide in combination with the binary vector containing the recombinase [27]. To modulate the *N*-glycosylation profiles, binary vectors containing the cDNA of the mammalian genes necessary for protein sialylation were co-infiltrated with the viral based vectors: namely, UDP-*N*-acetylglucosamine-2-epimerase/*N*-acetylmannosamine kinase (GNE), *N*-acetylneuraminic acid phosphate synthase (NANS), CMP-*N*-acetylneuraminic acid synthase (CMAS), CMP-sialic acid transporter (CST), and α2,6-sialyltransferase (ST) [29]. Agrobacteria carrying the magnICON® constructs were infiltrated using OD_600_ 0.05–0.2 and those carrying the other binary constructs were infiltrated using OD_600_ 0.05 (1.0 OD_600_ corresponds to 5 × 10^8^ cells/mL).

### 2.3 Purification of ^Sia^rhEPO

#### 2.3.1 2F5 immunoaffinity chromatography

Affinity chromatography was performed on a 2F5 antibody affinity column using the ÄKTAPurifier-10 FPLC system (GE Healthcare). 5 mg of purified 2F5 antibody (kindly supplied by Dr. Florian Rücker, Department of Biotechnology, University of Natural Resources and Life Sciences, Vienna, Austria) were coupled to a 1 mL pre-packed NHS-activated high performance Sepharose column (GE Healthcare) according to the manufacturer's instructions. Infiltrated leaf material (20 g) was ground in liquid nitrogen and thawed in an equal volume v/w of ice-cold extraction buffer (100 mM Tris-HCl pH 7.4, 30 mM ascorbic acid, 0.9% NaCl). The slurry was centrifuged (35 000 *g* for 30 min at 4°C) and the supernatant was vacuum filtrated (Macherey-Nagel filter circles MN619eh, 2–4 μm). The filtrate was further clarified by isoelectric precipitation at pH 4.5 (precipitation of ∼25% of total soluble proteins (TSPs) including RuBisCO), centrifuged as above and after adjustment to pH 7.4 incubated on ice for 15 min. Prior to loading on the chromatography column, the extract was passed through a 0.45 μm filter (Millipore Stericup Durapore low binding membrane PVDF, 73 mm/0.45 μm). The column was washed with 10 column volumes of binding buffer (PBS, pH 7.4) and the sample was applied onto the column at a flow rate of 0.9 mL/min. After washing with binding buffer, proteins were eluted with 50 mM glycine/HCl pH 2.8 and immediately neutralized with 0.5 M Tris. Column washing and elution were carried out at a flow rate of 1 mL/min. Prior to SDS-PAGE analysis the samples were precipitated in ice-cold acetone (4× sample volume) for 1 h at –20°C and centrifuged for 10 min at 16 000 *g*. The precipitate was air-dried, resuspended in 3× Laemmli buffer and heated at 96°C for 8 min.

#### 2.3.2 SNA lectin affinity chromatography

Affinity chromatography was performed using the ÄKTAPurifier-10 FPLC system on a 1.6 mL SNA lectin affinity column (GALAB Technologies GmbH). The ^Sia^rhEPO^ELD^-containing eluates from the 2F5 immunoaffinity chromatography were identified by dot blot using anti-hEPO antibodies and pooled. The sample was diluted with 2×SNA lectin binding buffer (40 mM Tris-HCl, 300 mM NaCl, 2 mM MnCl_2_, 2 mM CaCl_2_ 2 mM MgCl_2_, pH 7.0) and slowly (0.4 mL/min) applied to the column using a P1 peristaltic pump (GE Healthcare). Sample loading and column washing steps were performed at 4°C. The binding fraction was eluted with 0.3 M lactose in 50 mM glycine/HCl pH 3.2 buffer at a flow rate of 0.8 mL/min at room temperature. Subsequently the eluates were analyzed by dot blot with anti-hEPO antibodies and the positive fractions were pooled. The buffer was exchanged by passing the sample through an LC-4 solid phase extraction (SPE) column (Supelclean – Supelco), washing it with water and eluting it in 2 mL of 60% ACN + 0.1% TFA. After drying in the speed-vac the samples were resuspended in water.

### 2.4 SDS-PAGE and immune-based analyses

Recombinant proteins fractionated by 12.5% SDS-PAGE were either stained with CBB G-250 [31] or used for western blot analysis. Those used for immunoblot analysis were blotted to Hybond-ECL® nitrocellulose membranes (Amersham), blocked for 1 h in 1×PBS supplemented with 3% w/v BSA and 1% v/v Tween 20 and detected using either anti-Strep II (IBA GmbH, Germany, 1:2000 dilution in PBST + 3% BSA), anti-hEPO (R&D systems, 1:3000 dilution in PBST), or 2F5 (1 μg 2F5/mL in PBS + 1% BSA) antibodies. The dot blot was prepared by spotting 10 μL of sample onto the nitrocellulose membrane and blocking for 1 h, and was detected with anti-hEPO antibodies as above.

Quantification of rhEPO was carried out by a double sandwich ELISA using mouse monoclonal hEPO antibodies (Genzyme, Art. Nr. AE-7A5) for coating (dilution 1:10.000) and biotinylated (according to GE healthcare, RPN2202) polyclonal rabbit hEPO Abs (R&D Systems; Art. Nr. AB-286-NA; 1:800 diluted) as a second antibody. Streptavidin-peroxidase (Boehringer Mannheim, Art. Nr. 1 096 044) was used for detection. The color reaction was measured by an ELISA Reader at 492/620 nm. The CHO-derived rhEPO standard was provided by Polymun Scientific GmbH (Vienna).

### 2.5 Profiling of *N*-glycopeptides

*N*-glycan analysis of rhEPO was carried out by LC-ESI-MS of tryptic glycopeptides as described [32]. Briefly, the purified samples were submitted to reducing SDS-PAGE and the immunoreactive bands were cut from the gel, S-alkylated, double-digested with trypsin and endoproteinase Glu-C, eluted from the gel fragment with 50% acetonitril and separated on a Biobasic C18 column (150 mm × 0.32 mm, Thermo Electron) with a gradient of 1–80% acetonitrile containing 65 mM ammonium formate, pH 3.0. This double digestion allows the discrimination of all three rhEPO glycopeptides:glycopeptide 1:E^21^AENITGCAE^31^; glycopeptide 2:H^32^CSLNENITVPDTK^45^; and glycopeptide 3:G^77^QALLVNSSQPWEPLQHLVDK^97^. Positive ions were detected with a quadrupole-time of flight (Q-TOF) Ultima Global mass spectrometer (Waters, Milford, MA, USA). Summed and deconvoluted spectra of the glycopeptides elution range were used for identification of glycoforms.

### 2.6 In vitro activity assay of rhEPO

The in vitro biological activity of rhEPO^ELD^ was measured in an UT-7 cell-based proliferation assay. Briefly, the UT-7 cell line [33] was maintained in RPMI 1640 (Biochrome AG) supplemented with 10% fetal calf serum (PAN Biotech.), 4 mM L-glutamine and 5 ng/mL EPO. The cells were washed with EPO-free culture medium and incubated for 4 h at 37°C and 7% CO_2_. Increasing amounts of CHO-derived rhEPO and plant-derived rhEPO^ELD^ (ranging from 0.03 to 30 000 ng/mL) were added to 100 μL of medium containing about 10^4^ cells in a 96-well culture plate, resulting in a final rhEPO concentration of 0.01–10 000 ng/mL per well. After 4 days at 37°C and 7% CO_2_, 10 μL of an MTT (Thiazolyl Blue Tetrazolium Bromide; Sigma) solution (5 mg/mL) were supplied to each well and the plate was incubated for 4 h as before. Finally, 100 μL of 10% SDS (in 0.01 M HCl) were added to each well and mixed thoroughly at 37°C to dissolve blue crystals before absorbency was read at 570 nm (reference wavelength 690 nm). The results of the fivefold determination were evaluated using MS Excel Solver. The half-maximal effective UT-7 cell proliferation dose (ED50) was used to compare the activities of plant- and CHO-derived rhEPO.

## 3 Results

### 3.1 Vectors for rhEPO expression

Transient expression of rhEPO was achieved with magnICON® viral based vectors [27] designed to contain the respective cDNAs with a Strep II tag either at the N- or C-terminus (^Strep^rhEPO, rhEPO^Strep^, Fig. 1A). *Agrobacterium tumefaciens* suspension cultures carrying the recombinant plasmids were delivered to the expression host *N. benthamiana* by agroinfiltration. Infiltrated leaves were harvested 5 days post-infiltration (dpi) and expression of the recombinant hormone was monitored by western blot analysis of TSPs. Antibodies against hEPO exhibited a strong signal at the expected size of 30-kDa for both constructs; however, no signal was detected when antibodies against the Strep II tag were used (data not shown). These results indicate that either the tag is cleaved off or it is not accessible at either of the termini. In search for an alternative purification strategy we used an immunoaffinity-based purification with the epitope ELDKWA (derived from HIV-1-gp41) in combination with the corresponding mAb 2F5 [34]. hEPO constructs with a N- or C-terminal tag (^ELD^rhEPO, rhEPO^ELD^, Fig. 1A) were delivered to *N. benthamiana* and western blot analysis of TSP at 5 dpi showed strong signals at the expected size with both anti-hEPO and 2F5 antibodies (Supporting information Fig. S1). Consequently, we used the C-terminal-tagged rhEPO construct (rhEPO^ELD^) in subsequent experiments.

### 3.2 Transient expression of rhEPO^ELD^ in *N. benthamiana*

Various infiltration experiments were carried out to explore the maximum expression levels of rhEPO^ELD^ in *N. benthamiana*. The OD_600_ of agrobacterial suspension was varied (0.5–0.05 OD_600_) and leaves were infiltrated at different developmental stages. In addition, timecourses were implemented. Western blot analyses revealed that the optimum rhEPO^ELD^ expression could be achieved upon infiltration of middle-aged leaves (from plants that carry six to eight leaves) using agrobacteria at a concentration of OD_600_ 0.2. A timecourse from 2 to 11 dpi showed an expression maximum between 4 and 5 dpi (Fig. 1B). Western blot analysis with anti-hEPO antibodies showed a strong signal at the expected size of 30-kDa. In addition, two other bands were detected at positions 26- and 22-kDa (Fig. 1B). CHO-derived rhEPO was detected as an ∼40-kDa band (Fig. 1B, right panel C1, C2). This size discrepancy between plant- and CHO-derived rhEPO is most probably due to differences in *N*-glycosylation. The expression level of rhEPO, as determined by ELISA, varied from 50 to 85 mg rhEPO/kg fresh leaf material depending on the OD_600_ and dpi. With an OD_600_ of 0.2, a maximum expression level of 85 mg rhEPO/kg fresh leaf material was detected at 4 dpi, corresponding to ∼2% of TSP.

**Figure 2:**
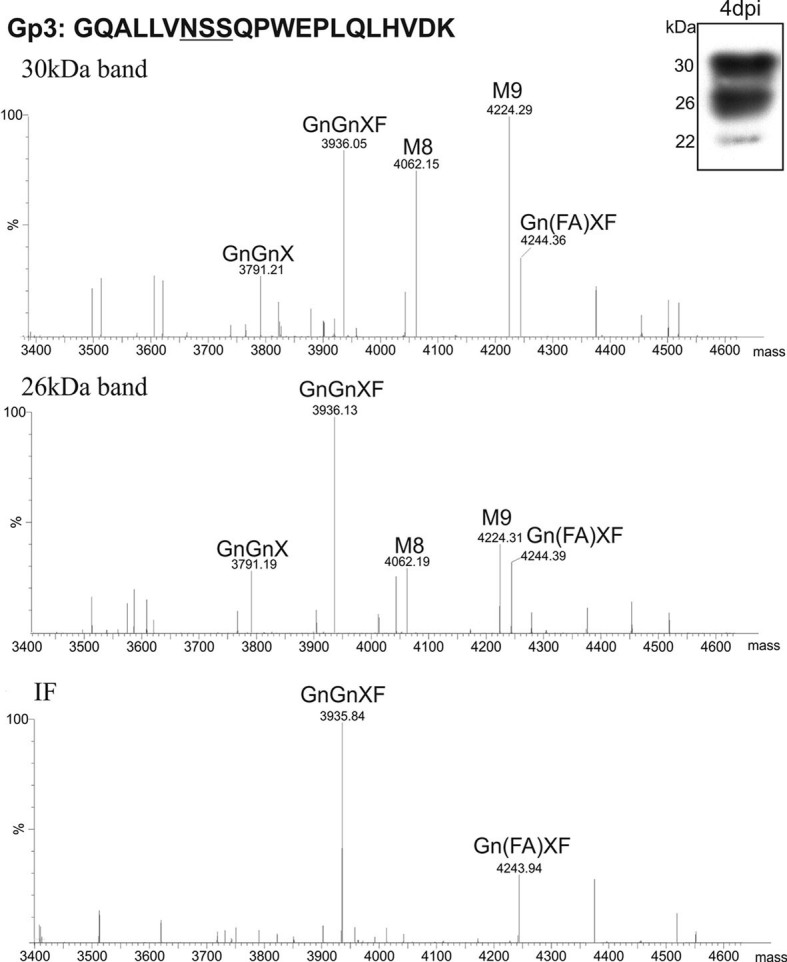
*N*-glycan profiles of rhEPO^ELD^ derived from *N. benthamiana* WT plants harvested at 4 dpi. At this time point western blot analysis of TSPs with anti-hEPO antibody shows three reactive bands (22-, 26-, and 30-kDa, top right cartoon). The *N*-glycan analysis was carried out by LC-ESI-MS of glycopeptides obtained by double digestion as described in Section 2. This allows discrimination of the three EPO glycopeptides (Gp1-Gp3). The MS profile of glycopeptide 3 (Gp3) is shown (see Supporting information Fig. S2 for analysis of glycopeptide 1 and 2). Top: profile of the 30-kDa band; middle: profile of the 26-kDa band; bottom: MS profile from rhEPO^ELD^ isolated from the IF. Two independent repeats of the experience were performed. Peak labels were made according to the ProGlycAn system (www.proglycan.com).

LC-ESI-MS analysis was performed to determine the *N*-glycosylation status of rhEPO^ELD^ expressed in WT *N. benthamiana* plants (^WT^rhEPO^ELD^). Corresponding bands (22-, 26-, and 30-kDa) were excised from denaturing gels loaded with TSP and double digested with trypsin and endoproteinase Glu-C to release three *N*-glycopeptides (Gp1-Gp3). Subsequent MS analysis revealed that all three *N*-glycosylation sites of ^WT^rhEPO^ELD^ carried a similar glycosylation pattern (Fig. 2 and Supporting information Fig. S2). The 26-kDa protein had a dominant single glycoform, i.e. GnGnXF, typical for plant-secreted proteins. This oligosaccharide formation was also present in the 30-kDa protein, however, the major fraction referred to ER-typical oligomannosidic structures (M8 and M9). Non-fucosylated complex glycans (GnGnX) and glycans containing galactose residues were detected in both bands. The galactosylated structures are most likely Lewis-a (Le^a^) carbohydrates (Gn(FA)XF), which have been previously reported on rhEPOFc expressed in *N. benthamiana* [35, 36] and are highly abundant on moss-derived rhEPO [24, 25]. MS analysis of the 22-kDa protein revealed non-glycosylated ^WT^rhEPO^ELD^, which accounts for less than 5% of the recombinant hormone. The differences in size upon gel electrophoresis are most probably associated with different glycosylation of the recombinant protein. Signals in immuno-blot analysis with 2F5 mAbs, specific for the ELD-Tag, indicate intact N- and C-termini of the recombinant protein (Supporting information Fig. S1). rhEPO^ELD^ was abundantly present in intercellular fluid (IF), indicating efficient secretion of rhEPO^ELD^ to this compartment. Glycan analysis of secreted rhEPO^ELD^ displayed a homogenous profile of plant-specific complex *N*-glycans (GnGnXF) and no significant amounts of oligomannosidic structures were detected (Fig. 2).

### 3.3 Modulation of rhEPO^ELD^ *N*-glycosylation

**Figure 3:**
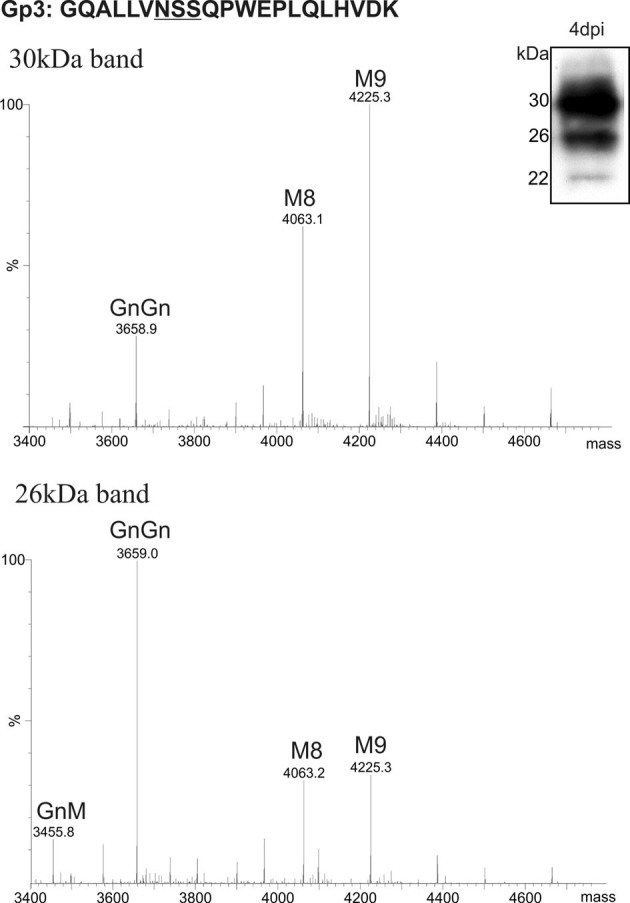
*N*-glycan profiles of rhEPO^ELD^ derived from *N. benthamiana* ΔXTFT harvested at 4 dpi. Western blot analysis of TSP extracts with anti-hEPO antibody shows three reactive bands (22-, 26-, and 30-kDa, top right cartoon). The glycosylation profile for glycopeptide 3 (Gp3) is shown. Top: MS profile corresponding to the 30-kDa band; bottom: MS profile corresponding to the 26-kDa band. Four independent repeats of the experience were performed. Peak labels were made according to the ProGlycAn system (www.proglycan.com).

We used *N. benthamiana* ΔXTFT as a host in a first set of experiments aimed at generating rhEPO^ELD^ with human-like *N*-glycosylation, i.e. lacking plant specific core α1,3-fucose and β1,2-xylose residues. The recombinant EPO (^ΔXTFT^rhEPO^ELD^) was produced in similar levels as ^WT^rhEPO^ELD^. Also, ^ΔXTFT^rhEPO^ELD^ was detected in three immunoreactive bands by western blot, exhibiting distinct glycosylation profiles: the 30-kDa protein band carried GnGn structures accompanied by high levels of oligomannosidic *N*-glycans (M8, M9), the 26-kDa band mainly carried complex GnGn structures and the 22-kDa band corresponded to non-glycosylated ^ΔXTFT^rhEPO^ELD^ (Fig. 3). No *N*-glycans carrying core α1,3-fucose or β1,2-xylose residues were detected. ^ΔXTFT^rhEPO^ELD^ showed a largely homogeneous glycosylation profile.

**Figure 4:**
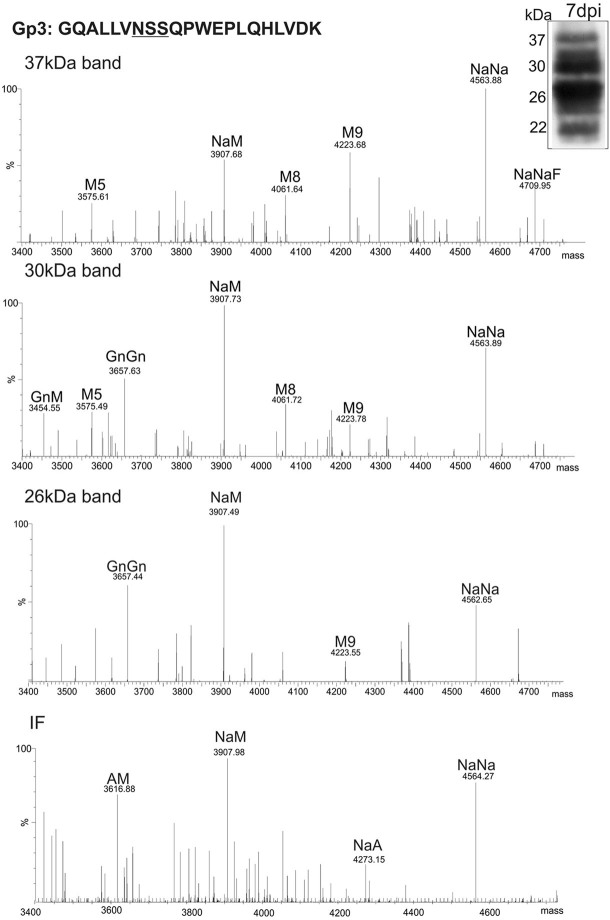
*N*-glycan profiles of rhEPO^ELD^ co-expressed with genes necessary for *in planta* sialylation in *N. benthamiana* Gal^+^, harvested at 7 dpi (^Sia^rhEPO^ELD^). Western blot analysis of TSP extracts with anti-hEPO antibody shows several reactive bands (22-, 26-, 30-, and 37-kDa, top right cartoon). The glycosylation profile (Gp3) of 37-, 30-, and 26-kDa immunoreactive bands are shown; bottom: MS profile of ^Sia^rhEPO^ELD^ isolated from the IF. (see Supporting information Fig. S3 for analysis of glycopeptide 1 and 2). Two independent repeats of the experience were performed. Peak labels were made according to the ProGlycAn system (www.proglycan.com).

In a next step, we set out to generate rhEPO^ELD^ carrying sialylated *N*-glycans. Recently we showed *in planta* sialylation of recombinant mAbs through the overexpression of six genes involved in the mammalian sialic acid pathway [29]. We used *N. benthamiana* GalT^+^ as the expression host to accomplish sialylation in plant-derived rhEPO^ELD^. This glycosylation mutant synthesizes mammalian-type terminal β1, 4-galactosylated structures, providing the acceptor substrate for subsequent sialylation [30]. Thus, rhEPO^ELD^ was co-expressed in *N. benthamiana* Gal^+^ plants with mammalian genes required for the synthesis of sialic acid (GNE, NANS, and CMAS), its transport into the Gogi (CST) and its transfer to terminally β1,4-galactosylated *N*-glycans (ST) (see Section 2). Subsequently, western blot analysis with antibodies against hEPO was used to monitor the expression of the recombinant hormone (^Sia^rhEPO^ELD^, Fig. 1B, right panel). The expression maximum was reached at 4 dpi, however, it seems that ^Sia^rhEPO^ELD^ is more stable than ^WT^rhEPO^ELD^ over time (Fig. 1B, left panel). Moreover, immunoreactive bands shifted toward increased size over time (Fig. 1B), indicating accumulation of the sialylated recombinant hormone. Apart from the three reactive bands (22-, 26-, and 30-kDa) an additional 37-kDa band appeared at 6 dpi. A detailed *N*-glycan analysis of the proteins from individual bands revealed that the 22-kDa protein, which accounts for less than 5% of the recombinant hormone, was non-glycosylated. The three larger bands (26-, 30-, and 37-kDa) exhibited substantial fractions of sialylated *N*-glycans, with some variation in bi-sialylated (NaNa) and incompletely processed structures (NaM) (Fig. 4 and Supporting information, Fig. S3). In addition, significant amounts of GnGn and minor fractions of oligomanno-sidic structures (M8, M9) were also present. Notably, ^Sia^rhEPO^ELD^ derived from the apoplastic fluid was decorated almost exclusively with sialylated *N*-glycans (IF, Fig. 4).

### 3.4 Purification of ^Sia^rhEPO^ELD^

**Figure 5:**
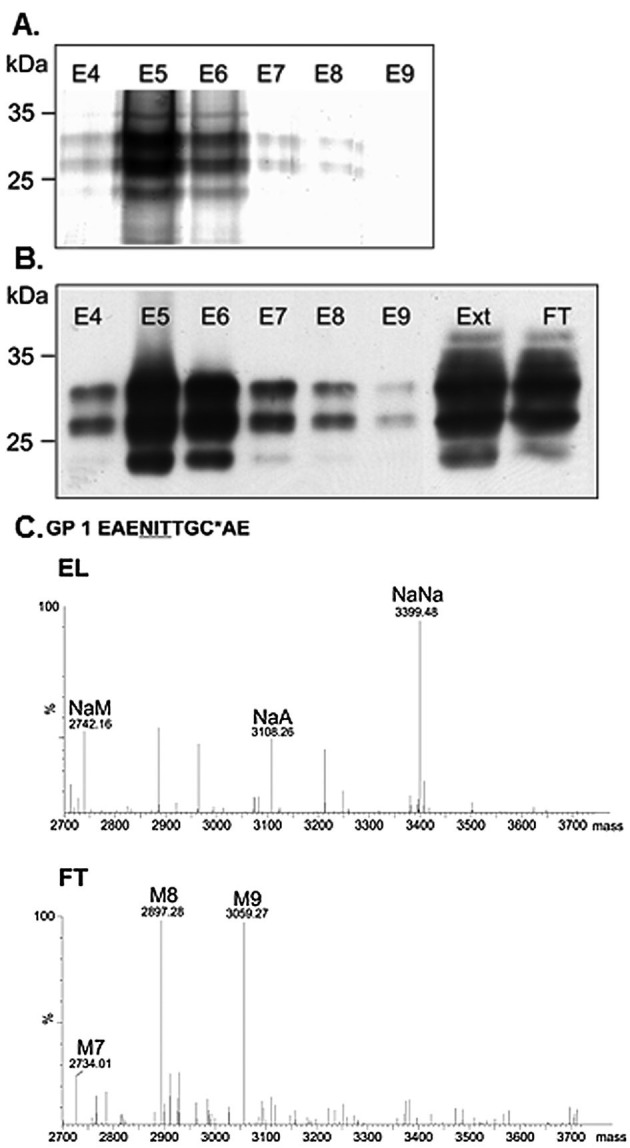
Analysis of 2F5 immunoaffinity-purified ^Sia^rhEPO^ELD^. (**A**) SDS-PAGE Coomassie-stained gel of eluates 4–9 (E4–E9; no protein quantification was made), (**B**) corresponding western blot using antibodies against hEPO, Ext: TSP extract, FT: FT after immunoaffinity chromatography (5 μg TSP was loaded). Several independent repeats of the experience were performed. (**C**) *N*-glycan profiles of ^Sia^rhEPO^ELD^ purified by sequencial 2F5 immunoaffinity and SNA lectin affinity chromatography (see also Supporting information Fig. S4). The glycosylation profile for glycopeptide 1 in the eluate (EL) and FT is shown. Peak labels were made according to the ProGlycAn system (www.proglycan.com).

Although the expression of rhEPO has been reported to be successful in various expression hosts, purification of the recombinant hormone remains a challenge. Previous attempts using a C-terminal His or Strep II tag to purify rhEPO proved unsuccessful. Although the expression levels of the hormone were similar to those obtained in this study, we were not able to purify the recombinant protein due to unspecific binding (His-tag) or loss of tag (Strep II tag) (our laboratory, unpublished data). In this study we explored immunoaffinity of the ELDKWA epitope to the mAb 2F5 [34] in view of establishing a new purification method. Five milligram of purified CHO-derived 2F5 were coupled to an NHS-Sepharose pre-packed chromatography column. Clarified leaf protein extracts from ^Sia^rhEPO^ELD^ were loaded onto the column and eluted at different pH conditions. A sharp elution peak was visible in eluates E5 and E6. These two eluates, with a total volume of 1 mL, contained about 90% of the purified product. We used CBB G-250 to detect the purified hormone as rhEPO is more sensitive to Brilliant Blue R-250 [31]. As already seen in immunoblots (Fig. 1B), the Coomassie-stained gels of purified recombinant hormone shows three protein bands (Fig. 5A) that react with anti-hEPO antibodies (Fig. 5B). Coomassie-stained gels upon loading 0.3 mL out of 0.5 mL eluate showed alongside the three major rhEPO specific bands, minor contamination with plant proteins (Supporting information, Fig. S4). Moreover significant amounts of the recombinant protein were detected in the flow through (FT, Fig. 5B), indicating that there is still room for optimization of the purification procedure. The *N*-glycosylation profile of the purified products largely resembled those observed in TSP extracts, where ^Sia^rhEPO^ELD^ is produced as a mixture of glycoforms including sialylated and high mannosidic structures. In our attempts to enrich the fraction of rEPO^ELD^ carrying sialylated glycoforms, we performed SNA lectin chromatography as a second purification step. A schematic representation of the experimental procedure is outlined in Supporting information Fig. S5. Briefly, samples from ^Sia^rhEPO^ELD^ purified by 2F5-based immunoaffinity were applied to the SNA lectin column, and elulates containing the recombinant protein were pooled and passed through an LC4 SPE reversed phase column to remove lactose. Subsequent *N*-glycosylation analysis of the product obtained revealed the presence of virtually exclusively sialylated *N*-glycan structures. An effective enrichment of bi-sialylated fractions accompanied by minor fractions of monoantennary sialylated structures was obtained (NaA, NaM, Fig. 5C, EL). As expected, rhEPO^ELD^ present in the FT carried exclusively oligomannosidic structures (Fig. 5C, FT), demonstrating the efficiency of the procedure. Note that although the SNA chromatography allowed isolation of fully sialylated rhEPO^ELD^, this step caused a significant reduction in product yield, and thus may to be optimized.

### 3.5 In vitro activity assay

The in vitro biological activity of plant-derived rhEPO^ELD^ was evaluated by a UT-7 cell-based proliferation assay and compared with CHO-derived sialylated rhEPO. The latter consisted mainly of tri- and tetrasialylated core fucosylated *N*-glycans (^CHO^rhEPO, Supporting information Fig. S6). Growth of the human leukemia cell line UT-7 is strictly correlated to the concentration of EPO in the culture medium. Cell-based in vitro assay indicated that plant-derived rhEPO^ELD^ was active (Supporting information, Fig. S7). ^Sia^rhEPO^ELD^ and ^CHO^rhEPO showed similar half maximum effective doses (ED50 values 0.66 and 0.53 ng/mL, respectively). As expected, non-sialylated ^WT^rhEPO^ELD^ exhibited increased activity (ED50 0.03 ng/mL) compared with its sialylated form.

## 4 Discussion

Erythropoietin is a multifunctional molecule produced and used by many tissues. Apart from its erythropoiesis action, other roles involve the acute and sub-acute biological responses to tissue damage [3, 4]. The extensive glycosylation of the protein is well characterized, and attempts have been made to generate hyperglycosylated forms of the protein in the course of developing drugs for treating anemia-associated conditions. However, considering the large *N*-glycan heterogeneity and multifunction of the hormone other glycoforms may be needed for new therapeutic applications [3, 4]. In the current study, we demonstrate the efficient expression of sialylated rhEPO^ELD^ in *N. benthamiana*, already at 4 dpi. This production speed provides advantages over other potential expression systems, such as mammalian cells. Terminal sialylation of rhEPO^ELD^ seemed to have a positive impact on the stability compared with ^WT^rhEPO^ELD^. Expression levels of up to 85 mg recombinant hormone/kg fresh leaf, corresponding to ∼2% of TSP, were achieved. Compared to previous studies where up to 0.02% expression levels of rhEPO in plants were reported [37] our results demonstrate a significant improvement. Nevertheless the expression of rhEPO^ELD^ was relatively low compared with expression levels of other recombinant proteins using similar transient production systems, e.g., monoclonal antibodies are produced up to 50% of TSP [38–41]. Similarly, the expression of a recombinant EPO fusion (rhEPOFc) using the same magICON® expression system resulted in the generation of a chimeric recombinant hormone accompanied by a five to ten times higher level of free Fc [35, 36]. The main goal of this study was to generate rhEPO^ELD^ carrying different *N*-glycosylation profiles. With this intent, WT *N. benthamiana* and the glycosylation mutants ΔXTFT and GalT^+^ were used as expression hosts. rhEPO^ELD^ derived from WT (^WT^rhEPO^ELD^) was decorated mainly with plant-specific GnGnXF^3^ structures. These are the typical *N*-glycan formations expected on secreted plant glycoproteins. However, in addition significant amounts (ca. 30%) of oligomannosidic structures and Le^a^ epitopes (ca. 15%) were detected. The presence of oligomannosidic structures was unexpected, they had not been detected previously in either native EPO or rhEPO expressed in mammalina cells [9, 11]. The fact that these structures were absent on IF-derived ^WT^rhEPO^ELD^, indicates that fractions of the protein are not properly secreted. It seems that part of rhEPO^ELD^ is sorted away from the secretory pathway and deposited in ER-derived compartments, a phenomenon observed previously upon expression of recombinant single chain antibodies in Arabidopsis seeds and *N. benthamiana* leaves [42, 43]. Le^a^ epitopes were another *N*-glycan formation detected on ^WT^rhEPO^ELD^ but not on native hEPO or CHO-derived rhEPO. Usually this glycoform accounts for only minor fractions of endogeneous *Nicotiana* proteins [28] and it had not been previously detected on plant-produced recombinant proteins [19, 20]. An exception is rhEPO expressed in moss cells, where large amounts of such oligosaccharide structures are present [25]. Le^a^ structures have also been reported on a rhEPOFc fusion expressed in *N. benthamiana* [35, 36]. The reason for plant-derived rhEPO carrying relatively large amounts of Le^a^ structures is currently not known.

*N*-glycans of rhEPO^ELD^derived from ΔXTFT (^ΔXTFT^rhEPO^ELD^) were essentially devoid of β1,2-xylose and core α1,3-fucose. Mainly two carbohydrate structures were present: complex GnGn and oligomannosidic structures. Surprisingly, no Le^a^ epitopes were detected. Finally, using *N. benthamiana* GalT^+^ as the expression host, an efficiently sialylated glycoform was produced (^Sia^rhEPO^ELD^) by coexpressing hEPO^ELD^ with the genes necessary for *in planta* sialylation. Moreover, sialylation seemed to have a stabilization effect on the recombinant hormone as ^Sia^rhEPO^ELD^ accumulated up to 9 days after infiltration, contrasting with the gradual reduction over time of ^WT^rhEPO^ELD^ expression.

Mammalian cell produced rhEPO is usually purified by ultrafiltration followed by ion exchange chromatography, reversed phase chromatography and gel filtration. These techniques are hardly applicable to crude plant leaf extracts. We evaluated different immunoaffinity tags in our attempts to establish an efficient purification protocol for plant produced rhEPO. A C-terminal His-tagged rhEPO showed similar expression levels to rhEPO^ELD^ but we were not able to efficiently purify the recombinant protein due to unspecific binding of the abundant endogeneous protein RuBisCO to Ni-Sepharose (our unpublished data). Another widely used tag (Strep II tag) failed as well because the tag was not detectable in western blot analysis, even though the hormone was nicely expressed. While searching for alternatives, we came across the mAb 2F5, which strongly binds to the epitope ELDKWA localized at the HIV-1 envelope protein gp41 [34]. We established a new immunoaffinity protocol that allowed purification of the recombinant hormone at great homogeneity. The sequencial immunoaffinity- and lectin affinity chromatography procedure allowed the enrichment of biantennary sialylated plant-derived rhEPO^ELD^. Nevertheless, this procedure, particularly SNA treatment, caused a massive loss of the product, and finally only ca. 5% of the amount present in TSP could be recovered. The applicability of 2F5-ELDKWA-based immunoaffinity purification at industrial scale still needs to be evaluated and optimized. Previous studies reporting high level of 2F5 expression in CHO cells are an excellent basis for mass production of the mAb at reasonable costs [44, 45]. The fact that the immunoaffinity column could be reused several times indicates high stability of the 2F5-Sepharose. Finally, we demonstrated full activity of the plant-derived hormone. The degree of sialylation does not seem to influence receptor binding, as seen by similar binding affinities of bisialylated ^Sia^rhEPO^ELD^and ^CHO^rhEPO, which mainly consists of tri- and tetrasialylated structures. On the other side, a higher receptor binding affinity of non-sialylated rhEPO^ELD^ was observed, a phenomenon already described for hEPO [46]. These results suggest a targeted manipulation of biological activities by alteration of the *N*-glycosylation profile. The results presented here together with the generation of branched *N*-glycosylated rhEPO in plants (tri and tetra-antennary structures, [35, 36] ) serve as an excellent starting point for the synthesis of multi-sialylated rhEPO, the preferred glycoform currently used in anemia treatment (work is in progress). Bisialylated hEPO is an abundant glycoform of anemia-patients derived serum EPO. The function of this glycosylation form is not known. Our results allow structure-function studies on this (and other) individual glycoprofile(s), and thus may help to better understand the action of this multifunctional hormone.

In summary the ability to generate different rhEPO^ELD^ with a targeted *N*-glycosylation profile paves the way to further investigate the impact of *N*-glycosylation on the function of the hormone, which in turn could lead to the discovery of new hEPO functions. Given the speed and ease with which different glycovariants of rhEPO^ELD^ were produced, the plant-based expression platform presented in this study is of great use not only in the research and development of pharmaceutical proteins but also in basic scientific investigations.

## Abbreviations

CBB: Coomassie brillant blue
CHO: Chinese hamster ovary
EPO: erythropoietin
FT: flow through
hEPO: human erythropoietin
IF: intercellular fluid
LC-ESI-MS: LC-electro-spray ionization-MS
rhEPO: recombinant human erythropoietin
SNA: *Sambucus nigra* agglutinin
TSP: total soluble protein
WT: wild type

## Supplementary material

As a service to our authors and readers, this journal provides supporting information supplied by the authors. Such materials are peer reviewed and may be re-organized for online delivery, but are not copy-edited or typeset. Technical support issues arising from supporting information (other than missing files) should be addressed to the authors.
