# Supplementary material for: Expression of functionally active sialylated human erythropoietin in plants
Source: Biotechnol J. 2013 Jan 17;8(3):371–82. doi: 10.1002/biot.201200363 (PMC3601435; doi:10.1002/biot.201200363)
Supplement: Supplementary file 1 [file biot0008-0371-SD1.pdf]

Biotechnology
Journal

Supporting Information for DOI 10.1002/biot.201200363

Expression of functionally active sialylated human erythropoietin in plants

Jakub Jez, Alexandra Castilho, Josephine Grass, Karola Vorauer-Uhl, Thomas Sterovsky, Friedrich Altmann and Herta Steinkellner

© 2013 Wiley-VCH Verlag GmbH & Co. KGaA, Weinheim

Supporting information

vector	Tag	Primer sequence 5`-3`
^{strep}rhEPO	N-WSHPQFEK	F:GCAGGTCTCAAGGTTGGTCACATCCTCAATTCGAAAAAGCTCCACCACGTCTTATC<br>R:GCAGGTCTCAAAGCCTAACGATCGCCAGTACGAC
rhEPO^{Strep}	C- WSHPQFEK	F:GCAGGTCTCAAGGTGCCCCACCACGCCTCATC<br>R: GCAGGTCTCAAAGCTCACTTTTCGAACTGCGGATG
^{ELD}rhEPO	N- ELDKWA	F:GCAGGTCTCAAGGTGAATTGGATAAGTGGGCTCCACCACGTCTTATC<br>R:GCAGGTCTCAAAGCCTAACGATCGCCAGTACGAC
rhEPO^{ELD}	C- ELDKWA	F:GCAGGTCTCAAGGTGCCCCACCACGCCTCATC<br>R: GCAGGTCTCAAAGCCCACTTATCCAATTCATCGCCAGTACGAC

Table S1: Sequence of the primers used in this investigation for the generation of magICON®3` modules carrying rhEPO with different peptide tags.

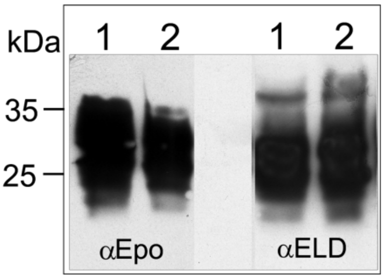

Figure S1: Western blot analysis was used to evaluate the recombinant protein expression in plants with specific anti-tag (αELD) or anti-protein (αhEPO) antibodies. The total soluble protein extract strongly reacted with both antibodies showing that rhEPO is expressed in plants and that the ELDKWA tag is present at the *N*-terminus (1: ^{ELD}rhEPO) and at the C-terminus (2: rhEPO^{ELD}).

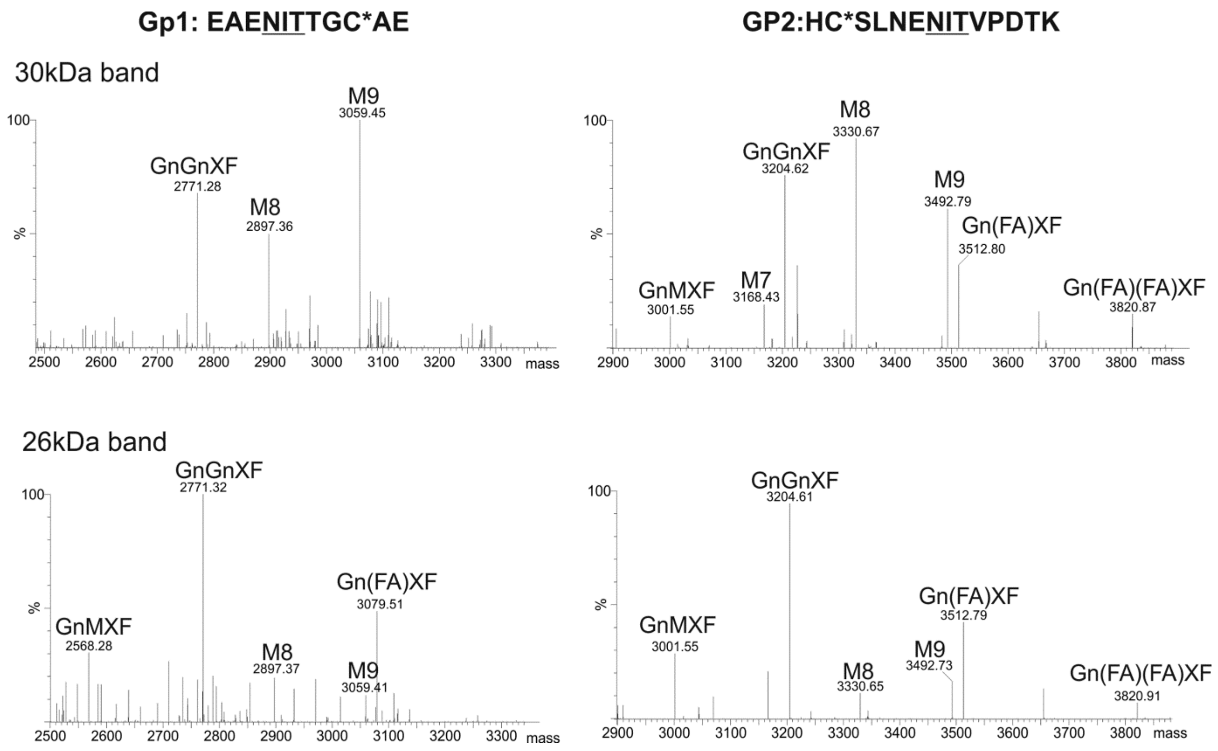

Figure S2: *N*-Glycan profiles of rhEPO^{ELD}expressed in *N. benthamiana* wild type plants (^{WT}rhEPO^{ELD}) at 4 days post-infiltration (dpi). The figure shows the glycosylation profile for both glycopeptide 1 and 2 in the 30- and 26-kDa bands detected by Western blot. Peak labels were made according to the ProGlycAn system (www.proglycan.com).

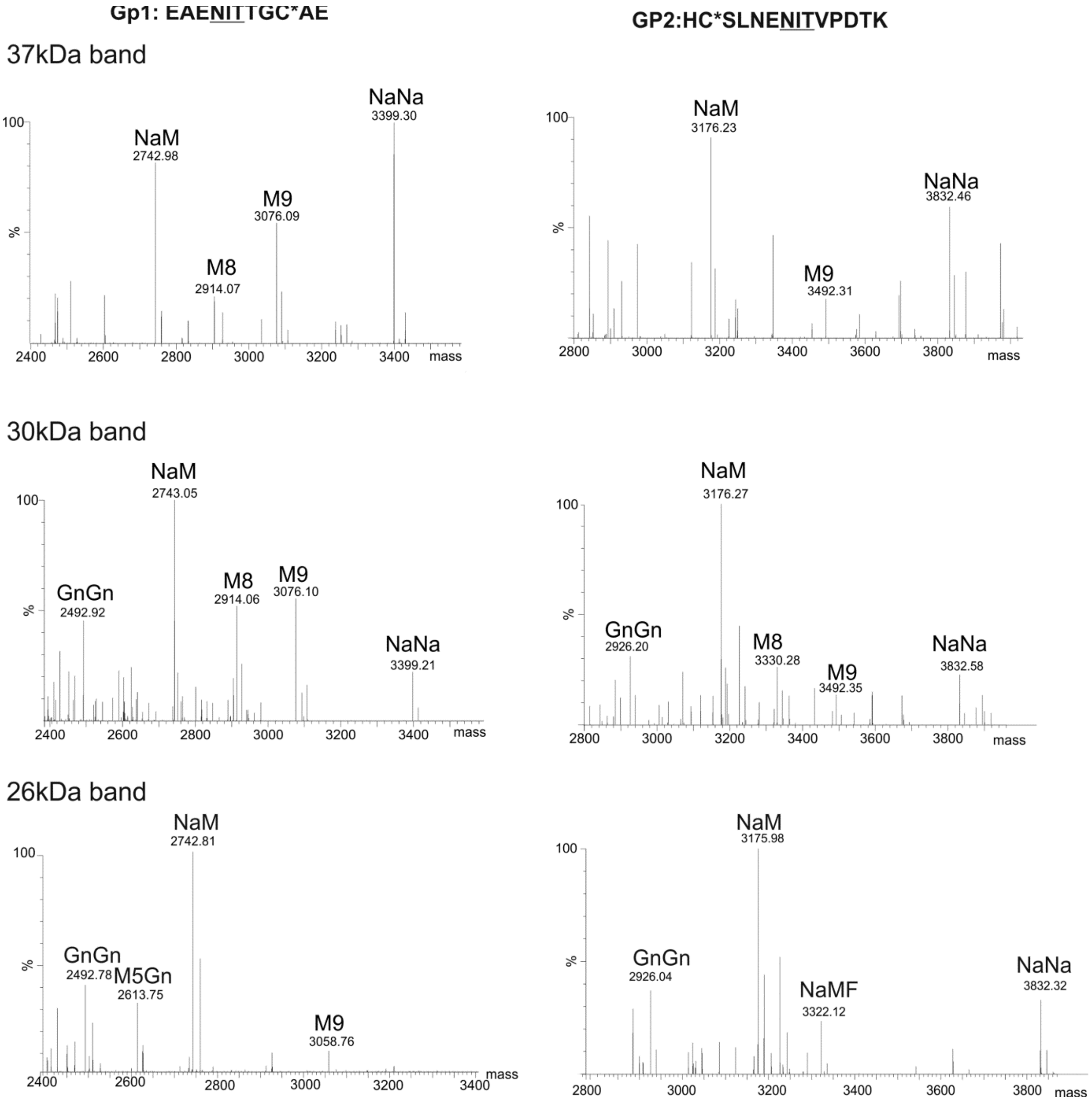

Figure S3: *N*-Glycan profiles of rhEPO^{ELD} co-expressed in *N. benthamiana* Gal^{+} with the genes for *in planta* sialylation and harvested at 7 dpi. The figure shows the glycosylation profile for glycopeptide 1 and 2 in the 37-, 30- and 26-kDa bands detected by Western blot. Peak labels were made according to the ProGlycAn system (www.proglycan.com).

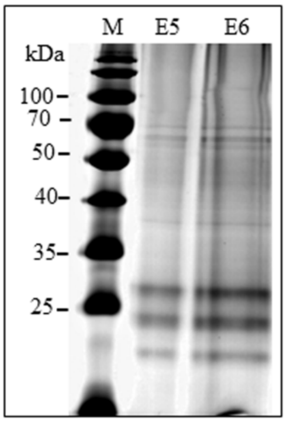

Figure S4: Coomassie stained gel of $^{Sia}rhEPO^{ELD}$ after one step 2F5 immunoaffinity purification. The figure shows the presence of three major bands on Eluates 5 and 6 as in Figure 5A (E5 and E6) with some faint bands most probably the result of contamination with plant proteins.

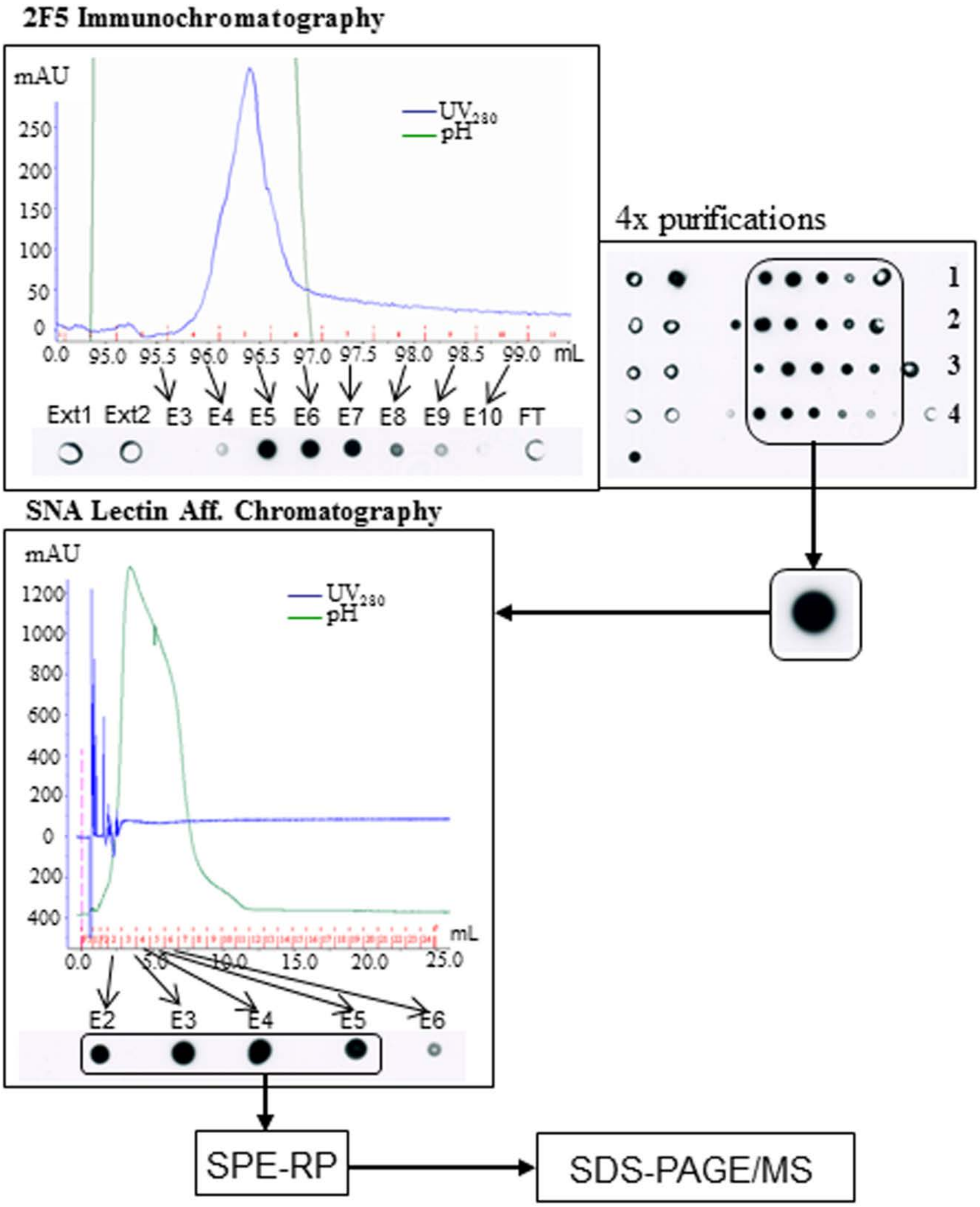

Figure S5: Schematic representation of a two-step purification protocol of ^{Sia}rhEPO^{ELD} expressed in *N. benthamiana* Gal^{+}mutants. A total of 80 g of infiltrated leaf material was used in 2F5 immunoaffinity chromatography. All eluates were analysed by dot blot with anti-hEPO antibodies. Eluates with positive signals were pooled and used in the second

purification step, SNA lectin affinity chromatography. The subsequent eluates were tested as before and again the positives were pooled and further purified in a LC-4 SPE column. After a final elution step the samples were fractionated by SDS-PAGE and analysed by Coomassie staining and Western blotting.

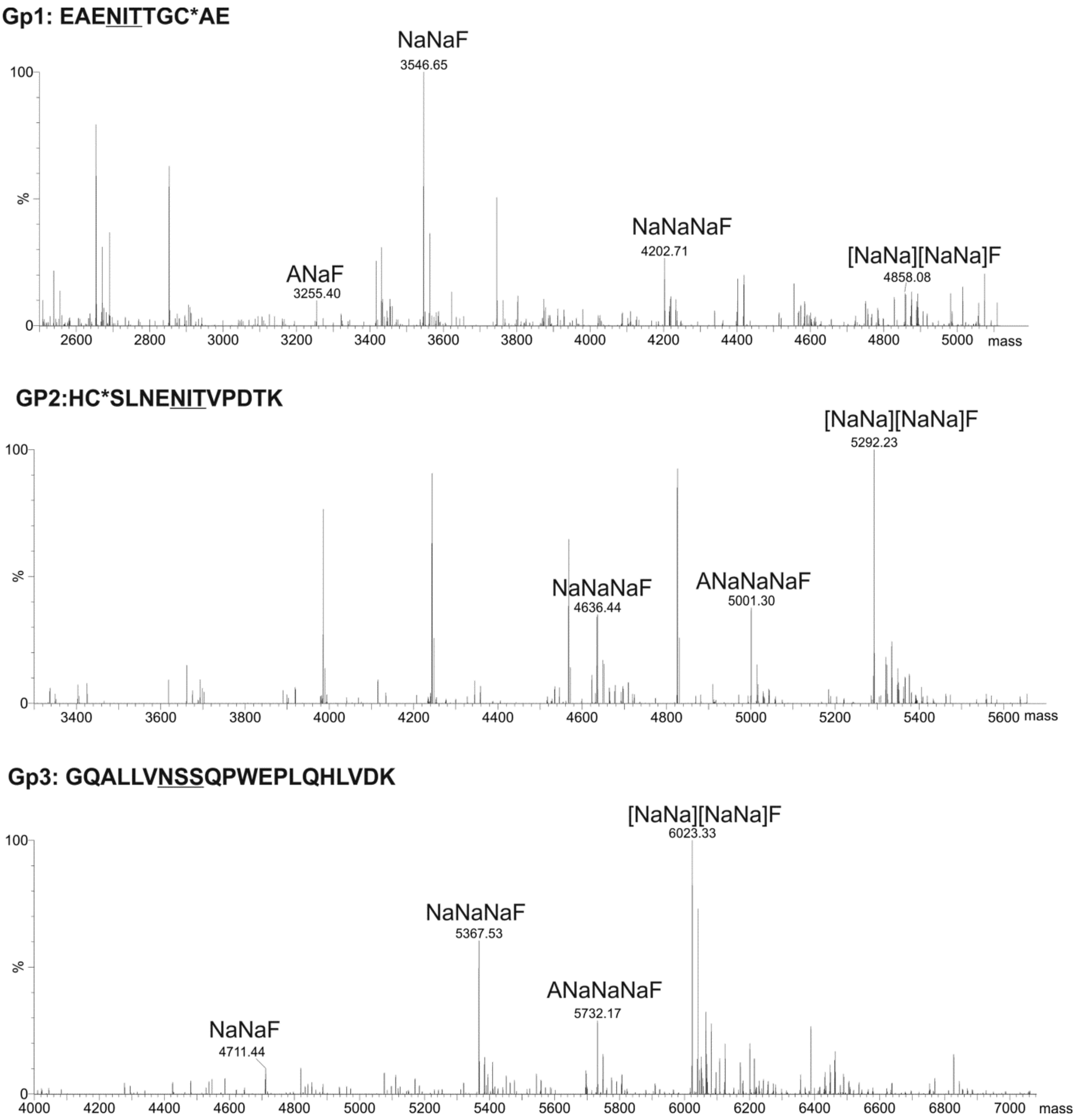

Figure S6: *N*-Glycan profile of CHO-derived rhEPO (all three glycopeptides). Peak labels were made according to the ProGlycAn system (www.proglycan.com).

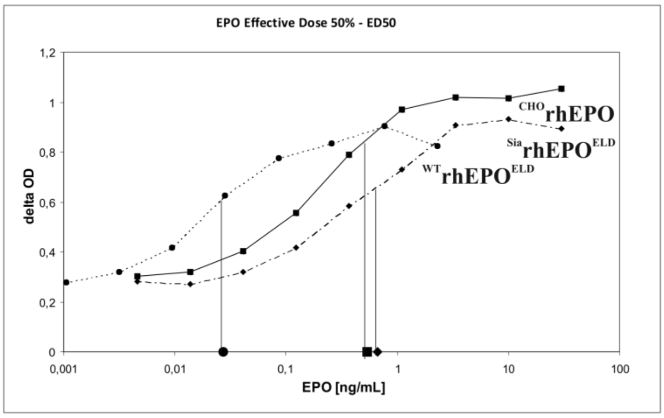

Figure S7: *In vitro* activity assay of plant-derived rhEPO^{ELD} and rhEPO derived from CHO cells. rhEPO half maximum effective doses (ED50) are displayed by vertical bars for the three samples: ^{Sia}rhEPO^{ELD}: 0.66 ng/mL; ^{CHO}rhEPO: 0.53 ng/mL and ^{WT}rhEPO^{ELD}: 0.03 ng/mL
